# On the Right Track

**Published:** 1891-03-07

**Authors:** 


### Passing Topics.

### ON THE RIGHT TRACK.

We think the hospitals and the Lords' Committee are alike to be congratulated upon the manifest improvement in the method of conducting the inquiry into the administration of medical relief in the metropolis, which has characterised the proceedings since the Committee resumed. We will not attempt to explain how so great a change has come about. Whether the remonstrances addressed from many quarters actually prevailed, or whether the Lords of their own inner consciousness began to realise that they were making but a poor display, and still poorer progress in regard to the weighty questions they had met to elucidate, it is needless to ask. Enough that the change has occurred, and that in place of aimless flounderings in a mire of petty personalities and feminine antipathies, we find that august body, collectively and individually, addressing themselves to the important issues involved in the undertaking they have engaged upon, and moving briskly forward as though they had at last become aware of the fact that they were charged with a better mission than to form a rallying point for everybody with a grievance.

The quality of the evidence recently given has been, upon the whole, satisfactory and to the purpose. The several secretaries who have been called have exhibited a creditable intimacy with the details of the working of their particular institutions, and an earnestness of interest for which it may be safely predicted outside critics were unprepared. It is not every man, of course, who could do himself or his institution full justice under a vivâ voce examination ranging over a wide field. It is an ordeal always fraught with peril; but, so far, we think, the cause of the hospitals has been well served by their chief officials, and the silliness of the talk about comfortable sinecures held at the expense of charity, in which a multitude of well-meaning but ignorant people have been wont to indulge, has been amply demonstrated.

Under the conditions which obtain in respect of the administration of medical charity, few offices are fraught with more onerous and more responsible duties, or require to be performed with greater care and tact than those of a hospital secretary; and, added to the inherent difficulties of the position, it is undeniable that in some cases, at least, there is an ungenerous hesitation on the part of committees to support the officer who is their representative and the embodiment of all lay authority. We believe this arises chiefly from a want of fuller knowledge. Few people but those who spend their lives in a hospital can realise the complexity of an organization in which the lay and medical elements are so inextricably blended. The pertinent questions put by the Chairman of the Committee and by several of its members, point to the conclusion that the Lords are becoming alive to certain difficulties of management which it has been too much the fashion to ignore. To the question what they would do in certain hypothetical but easily-reached circumstances, some of the secretaries have been unable to give more than a qualified and uncertain answer. Discipline is one of the most important elements in the administration of any institution, and if it should be established that the present methods are unequal to maintaining it continuously in our hospitals, we must hope that means will be found to construct an authority more fully adapted to the undoubted necessities of the case.

Agricola.

